# Redox Imbalance and Antioxidant Adaptation in Acute Ischemic Stroke: Temporal Changes in Enzymatic and Non-Enzymatic Markers

**DOI:** 10.3390/molecules31101767

**Published:** 2026-05-21

**Authors:** Jakub Garbarek, Julia Karolina Markiel, Wojciech Rzepka, Kamil Glazik, Magdalena Pitek, Karolina Szewczyk-Golec, Beata Kukulska-Pawluczuk, Natalia Soja-Kukieła, Alina Woźniak, Jarosław Nuszkiewicz

**Affiliations:** 1 Student Research Club of Medical Biology and Biochemistry, Department of Medical Biology and Biochemistry, Faculty of Medicine, Ludwik Rydygier Collegium Medicum in Bydgoszcz, Nicolaus Copernicus University in Toruń, 24 Karłowicza St., 85-092 Bydgoszcz, Poland; 312913@stud.umk.pl (J.G.); 316560@stud.umk.pl (J.K.M.); 316679@stud.umk.pl (W.R.); 316667@stud.umk.pl (M.P.); 2 Department of Medical Biology and Biochemistry, Faculty of Medicine, Ludwik Rydygier Collegium Medicum in Bydgoszcz, Nicolaus Copernicus University in Toruń, 24 Karłowicza St., 85-092 Bydgoszcz, Poland; karosz@cm.umk.pl; 3 Department of Neurology, Faculty of Medicine, Ludwik Rydygier Collegium Medicum in Bydgoszcz, Nicolaus Copernicus University in Toruń, 9 M. Skłodowskiej—Curie St., 85-094 Bydgoszcz, Poland; bkukulska@cm.umk.pl; 4 Centre for Statistical Analysis, Nicolaus Copernicus University in Toruń, Chopina 12/18 St., 87-100 Toruń, Poland; natalia.sk@umk.pl

**Keywords:** acute ischemic stroke, antioxidant enzymes, catalase, glutathione peroxidase, malondialdehyde, melatonin, oxidative stress, superoxide dismutase, vitamin D

## Abstract

Acute ischemic stroke (AIS) is associated with redox imbalance; however, the early temporal changes in enzymatic and non-enzymatic antioxidant responses are poorly understood. The aim of this study was to evaluate changes in selected oxidative stress markers during the early phase of AIS. The study was designed as a longitudinal within-subject analysis, with each patient serving as their own reference between day 1 and day 8. A total of 48 patients (mean age 69.31 ± 1.59 years; 56.3% male; mean body mass index (BMI) 27.05 ± 0.61 kg/m^2^), predominantly presenting with mild to moderate stroke severity, were enrolled in a prospective observational study. The cohort was characterized by a high prevalence of hypertension (87.5%), dyslipidemia (45.8%), and diabetes or prediabetes (45.9%). Blood samples were collected on day 1 and day 8 after stroke onset. Depending on the distribution of paired differences, either the paired Student’s *t*-test or Wilcoxon signed-rank test was applied. A significant increase in superoxide dismutase (SOD) activity was observed (1932.73 vs. 2086.55 U/g Hb, *p* = 0.032), whereas catalase (CAT; 403.19 vs. 415.30 × 10^3^ U/g Hb, *p* = 0.444) and glutathione peroxidase (GPx; 24.70 vs. 24.40 U/g Hb, *p* = 0.477) showed no significant changes. Similarly, malondialdehyde (MDA) levels remained stable in both erythrocytes (182.96 vs. 187.15 nmol/g Hb, *p* = 0.838) and plasma (0.41 vs. 0.41 nmol/mL, *p* = 0.922). In contrast, melatonin (59.65 vs. 55.49 pg/mL, *p* = 0.042) and 25-hydroxyvitamin D (25(OH)D; 19.31 vs. 16.52 ng/mL, *p* < 0.001) concentrations significantly decreased. These findings suggest that the early phase of AIS may be associated with a selective and potentially maladaptive antioxidant response, involving increased SOD activity alongside depletion of systemic modulators, which may contribute to persistent redox imbalance.

## 1. Introduction

Acute ischemic stroke (AIS) is one of the leading causes of mortality and long-term disability worldwide. With over 7.8 million new cases reported annually, AIS represents the most common type of stroke, accounting for approximately 65% of all incident cases [1]. AIS occurs as a result of vascular occlusion, which leads to a reduction in cerebral blood flow and subsequent deprivation of oxygen and glucose in the affected brain region. This results in mitochondrial dysfunction, disruption of cellular energy metabolism, and increased production of reactive oxygen species (ROS), ultimately leading to oxidative stress (OS) and neuronal injury [2,3].

Although ischemia is defined by oxygen deficiency, it paradoxically contributes to the generation of OS. Impaired mitochondrial electron transport, accumulation of reducing equivalents, and subsequent reperfusion lead to excessive ROS production [4]. Additionally, ischemia-induced ionic imbalance promotes the activation of various enzymes, including peroxidases, lipases, and nucleases, which further contribute to cellular damage and degradation of neuronal organelles [5]. Loss of mitochondrial membrane integrity results in the release of large amounts of free radicals, further exacerbating oxidative damage [6].

The brain is particularly vulnerable to OS due to its relatively low levels of endogenous antioxidant defenses and high content of polyunsaturated fatty acids (PUFAs), which are highly susceptible to peroxidation [7]. Excessive ROS production leads to lipid peroxidation, resulting in loss of membrane integrity and altered cellular permeability [8]. One of the most widely recognized end products of this process is malondialdehyde (MDA), which reflects the extent of lipid damage and is commonly used as a biomarker of OS in AIS [9].

The endogenous antioxidant defense system plays a crucial role in counteracting oxidative damage. Key enzymatic antioxidants include superoxide dismutase (SOD), catalase (CAT), and glutathione peroxidase (GPx) [10]. SOD catalyzes the conversion of superoxide anion (O_2_^●−^) into hydrogen peroxide (H_2_O_2_), which is subsequently detoxified to water (H_2_O) and oxygen (O_2_) by CAT or reduced to water with the simultaneous oxidation of glutathione (GSH) by GPx. Additionally, GPx is responsible for the reduction in other organic peroxides [11,12,13].

In addition to enzymatic antioxidants, endogenous modulators such as melatonin and vitamin D play important neuroprotective roles. Melatonin acts as a potent free radical scavenger and modulates the activity of antioxidant enzymes, including SOD [14]. Vitamin D is involved in the regulation of inflammatory and redox pathways, including inhibition of the nuclear factor kappa-light-chain-enhancer of activated B cells (NF-κB) signaling pathway and activation of the nuclear factor erythroid 2-related factor 2 (Nrf2)-dependent antioxidant response [15]. Although the antioxidant and neuroprotective effects of melatonin and vitamin D have been widely described, their dynamic interplay with enzymatic antioxidant systems during the course of AIS remains poorly understood. In particular, most previous studies have focused on individual biomarkers assessed at single time points, while comprehensive longitudinal analyses integrating both enzymatic and non-enzymatic components of the antioxidant system are lacking. This limits our understanding of the temporal coordination of redox responses in AIS and their potential role in disease progression and recovery.

The generation of ROS in AIS is a multifactorial process associated both with ischemia-induced metabolic disturbances and reperfusion-related oxidative bursts [4]. These processes lead to significant alterations in redox homeostasis, which are particularly detrimental to neurons due to their high metabolic activity and susceptibility to oxidative damage [16]. Therefore, evaluating the dynamics of endogenous antioxidant defense mechanisms during the course of AIS may provide important insights into the progression of oxidative injury [17]. Understanding these dynamic changes may provide a more comprehensive view of OS mechanisms in AIS and identify potential targets for therapeutic intervention.

In this study, we aimed to analyze early temporal changes in selected OS markers in patients with AIS between the 1st and 8th day after stroke onset. The analyzed parameters included the activity of antioxidant enzymes (SOD, CAT, and GPx), levels of MDA in erythrocytes (MDAer) and plasma (MDApl) as markers of lipid peroxidation, as well as concentrations of melatonin and 25-hydroxyvitamin D (25(OH)D).

## 2. Results

### 2.1. Characteristics of the Study Population

A total of 48 patients with AIS were included in the study. The majority of participants were male (56.3%), and the mean age of the study population was 69.31 ± 1.59 years. Most patients were classified as overweight or obese (62.6%), with only 29.2% presenting a normal body mass index (BMI).

Regarding vascular risk factors, hypertension was highly prevalent (87.5%), while diabetes was present in 27.1% of patients and prediabetes in 18.8%. Dyslipidemia was observed in 45.8% of participants. Most patients were non-smokers (60.4%), while 35.4% were current smokers.

In terms of stroke characteristics, the majority of patients presented with mild to moderate stroke severity on admission, as reflected by National Institutes of Health Stroke Scale (NIHSS) scores, with only a very small proportion presenting with severe stroke. Most patients had NIHSS scores in the range of 1–15 at baseline, with a similar distribution observed on day 8. This distribution was consistent with relatively high Alberta Stroke Program Early CT Score (ASPECTS) scores in most patients. According to the Trial of ORG 10172 in Acute Stroke Treatment (TOAST) classification, stroke of undetermined etiology was the most frequent subtype (54.2%), followed by small-vessel occlusion (18.8%) and cardioembolism (16.7%).

Regarding treatment, most patients (83.3%) did not receive reperfusion therapy, while thrombolysis was performed in 12.5% and thrombectomy in 4.2% of cases.

A detailed description of the study population is provided in the Section 4.

### 2.2. Changes in Oxidative Stress Markers Between Day 1 and Day 8

A statistically significant increase in SOD activity was observed over time. The mean SOD activity increased from 1932.73 ± 64.73 U/g hemoglobin (Hb) at baseline to 2086.55 ± 99.15 U/g Hb on day 8. Median values also increased from 1815.73 to 1893.86 U/g Hb, accompanied by broader interquartile ranges (586.53 vs. 876.81). The observed change was statistically significant (Wilcoxon signed-rank test, *p* = 0.032).

In contrast, no statistically significant changes were found for CAT activity. The mean CAT activity increased slightly from 403.19 ± 135.80 to 415.30 ± 132.79 × 10^3^ U/g Hb; however, this difference was not statistically significant (paired Student’s *t*-test, *p* = 0.444). Median values also showed a modest increase (392.90 vs. 425.00 × 10^3^ U/g Hb), while interquartile ranges decreased (136.43 vs. 118.40).

Similarly, GPx activity remained stable over time. The mean GPx activity changed from 24.70 ± 0.92 to 24.40 ± 0.92 U/g Hb. Median values remained comparable (23.95 vs. 24.48), with slightly increased interquartile ranges (5.90 vs. 6.56). The observed change was not statistically significant (Wilcoxon signed-rank test, *p* = 0.477).

No significant differences were observed in lipid peroxidation markers. The concentration of MDAer remained relatively unchanged (182.96 ± 12.20 vs. 187.15 ± 12.45 nmol MDA/g Hb, paired Student’s *t*-test, *p* = 0.838), with similar median values (197.13 vs. 198.14) and a moderate increase in interquartile range (126.22 vs. 152.85). Likewise, the concentration of MDApl showed no significant variation between time points (0.41 ± 0.01 vs. 0.41 ± 0.01 nmol MDA/mL, paired Student’s *t*-test, *p* = 0.922), with minimal changes in median (0.39 vs. 0.41) and interquartile range (0.12 vs. 0.09).

In contrast to enzymatic antioxidant parameters, both analyzed endogenous modulators demonstrated significant temporal changes. Melatonin levels significantly decreased from 59.65 ± 3.90 pg/mL at baseline to 55.49 ± 3.27 pg/mL on day 8 (paired Student’s *t*-test, *p* = 0.042). This decrease was also reflected in median values (55.60 vs. 50.14) and interquartile ranges (25.80 vs. 24.13).

A more pronounced reduction was observed for 25(OH)D. The mean concentration decreased from 19.31 ± 2.11 ng/mL to 16.52 ± 1.81 ng/mL. Median values declined from 16.33 to 13.94, accompanied by a decrease in interquartile range (16.76 vs. 12.08). The observed reduction was statistically significant (Wilcoxon signed-rank test, *p* < 0.001).

Changes in OS markers, antioxidant enzyme activities, lipid peroxidation indices, and endogenous modulators between day 1 and day 8 are presented in Table 1.

Correlation analysis revealed no significant associations between antioxidant enzyme activities (SOD, CAT, GPx) and NIHSS scores at baseline or on day 8. Similarly, changes in biomarker levels were not significantly correlated with changes in NIHSS.

## 3. Discussion

The present study provides a comprehensive evaluation of temporal changes in both enzymatic and non-enzymatic components of the antioxidant defense system during the early phase of AIS. Our findings demonstrate a distinct pattern characterized by a significant increase in SOD activity, accompanied by a lack of significant changes in CAT, GPx, and lipid peroxidation markers, alongside a significant decrease in endogenous modulators, including melatonin and 25(OH)D.

Importantly, these results suggest that the redox response in AIS is not uniform but rather reflects a selective and potentially dysregulated adaptation of the antioxidant system. The observed increase in SOD activity, in the absence of coordinated changes in downstream antioxidant enzymes, together with the simultaneous reduction in key systemic modulators, may indicate a selective antioxidant response accompanied by limited coordination between different components of the antioxidant system [18]. This pattern supports the concept that OS in ischemic stroke is not solely a consequence of excessive ROS production but also results from an imbalance between different components of the antioxidant defense network [19,20].

In the present study, we observed a statistically significant increase in SOD activity between day 1 and day 8 after stroke onset, whereas no significant changes were detected for CAT or GPx. This pattern suggests that the enzymatic antioxidant response during the early phase of AIS is not uniformly activated, but rather reflects a selective enhancement of superoxide-scavenging capacity.

The increase in SOD activity may represent an adaptive response to enhanced O_2_^●−^ generation associated with ischemia-induced mitochondrial dysfunction and subsequent reperfusion-related oxidative stress [21,22]. This interpretation is supported by previous reports demonstrating that SOD activity may rise during the subacute phase of ischemic stroke, particularly in erythrocytes [23]. In contrast, decreased SOD activity has been observed in serum in the acute phase of cerebral ischemic injury, with a gradual return toward control values during the following days [24]. Similarly, longitudinal studies indicate that antioxidant enzyme activity undergoes dynamic changes over time after ischemic stroke. In erythrocytes, both SOD and GPx activities have been reported to decrease in the early phase and subsequently recover to levels comparable to controls [25], whereas other studies have demonstrated increased SOD activity and, in some cases, elevated GPx activity at selected time points following AIS, suggesting activation of endogenous antioxidant defenses [26]. In this context, the elevation of SOD observed in our study likely reflects increased superoxide-scavenging activity in response to ischemia-associated oxidative stress during the early post-stroke period.

At the same time, the absence of significant changes in CAT and GPx activity suggests that this response may be incomplete or functionally unbalanced. Therefore, an increase in SOD activity without a parallel upregulation of downstream antioxidant enzymes may be associated with less efficient hydrogen peroxide detoxification and may contribute to persistent oxidative imbalance [27,28]. This pattern may therefore reflect an incomplete or insufficiently coordinated antioxidant response rather than a fully protective adaptation. Previous studies investigating antioxidant enzyme activity in acute ischemic stroke have reported heterogeneous and sometimes inconsistent findings, with variable changes observed for SOD, CAT, and GPx depending on the timing of measurement, stroke severity, and the biological material analyzed, which may be partially explained by differences in methodologies, sample size, and heterogeneity of study populations [29,30,31].

The lack of significant change in GPx activity in our cohort is particularly noteworthy, given that GPx has been described as a dynamic and potentially prognostically relevant antioxidant enzyme in acute ischemic stroke [32]. Both decreases and increases in GPx activity have been reported, depending on the stage of stroke and the characteristics of the study population [25,33,34,35]. Early reductions in GPx activity are often attributed to increased consumption of reduced glutathione (GSH) during acute oxidative stress, whereas subsequent increases may reflect compensatory upregulation of antioxidant defenses [25,33,34,35]. Some studies have linked higher GPx activity with lower neurological deficit severity and more favorable early outcomes, as reflected by lower NIHSS scores [25,32,33]. In this context, the relatively stable GPx activity observed in our study may indicate a balance between enzyme consumption and regeneration or a limited capacity for adaptive upregulation in the early post-stroke phase.

A similar interpretation may also apply to CAT, whose activity remained unchanged over time. Since both CAT and GPx are responsible for hydrogen peroxide detoxification, the lack of significant modulation of these enzymes suggests that the downstream antioxidant response may be less responsive than the initial superoxide-scavenging step. This may be particularly relevant in the context of AIS, where OS evolves dynamically and may involve sequential activation, exhaustion, or insufficient recruitment of different antioxidant pathways [17,36]. Importantly, previous studies have also highlighted the potential prognostic relevance of GPx activity, demonstrating inverse associations with neurological deficit severity and functional outcomes [33,37].

Overall, our findings suggest that the enzymatic antioxidant response in AIS is selectively activated rather than uniformly coordinated. The increase in SOD activity, in the absence of corresponding changes in CAT and GPx, may reflect an adaptive but incomplete antioxidant response. This pattern may also be related to the relatively homogeneous clinical profile of our cohort, composed predominantly of patients with mild to moderate stroke severity. More pronounced alterations in CAT and GPx activity may become apparent in cohorts including patients with more severe ischemic injury.

The lack of significant correlations between oxidative stress markers and stroke severity may suggest that early redox responses reflect systemic processes rather than being directly linked to neurological deficit as measured by NIHSS. Additionally, the relatively narrow range of NIHSS scores in our cohort, predominantly representing mild to moderate stroke, may have limited the ability to detect such associations.

In contrast to enzymatic antioxidant parameters, our study did not demonstrate significant changes in lipid peroxidation markers, as reflected by stable MDA concentrations in both erythrocytes and plasma between day 1 and day 8 after stroke onset. This finding may appear inconsistent with a substantial body of literature reporting elevated MDA levels in patients with AIS, particularly in association with more severe neurological deficits and unfavorable clinical outcomes [38,39,40].

Previous studies have shown that increased MDA concentrations are associated with stroke severity and may serve as a prognostic indicator. For instance, elevated serum MDA levels have been linked to worse outcomes, with significantly higher concentrations observed in patients with moderate to severe stroke compared to those with mild stroke and healthy controls [41]. These findings support the concept that lipid peroxidation plays a key role in the pathophysiology of ischemic brain injury and may reflect the extent of oxidative damage.

However, not all studies have demonstrated consistent elevations in MDA levels, particularly in the early phase of stroke or in populations with less severe neurological impairment. In the study by Maes et al. [42], conducted in patients with predominantly mild to moderate acute ischemic stroke, plasma MDA levels were not significantly different from those of controls, despite clear evidence of oxidative stress reflected by elevated lipid hydroperoxides and reduced antioxidant defenses. These findings suggest that MDA may not always be a sufficiently sensitive marker of early lipid peroxidation in less severe AIS and that different oxidative stress markers may exhibit distinct temporal profiles.

In contrast, earlier studies have demonstrated that MDA levels may increase over time following stroke onset. Sharpe et al. [43] reported no significant differences in MDA concentrations at admission, but a significant increase was observed after 48 h, accompanied by a decrease in ascorbate levels, suggesting consumption of antioxidant reserves and progression of lipid peroxidation. These findings support the concept that oxidative damage may evolve dynamically, with delayed manifestation of lipid peroxidation markers during the early post-stroke period.

Taken together, these observations indicate that the temporal changes in oxidative stress biomarkers are complex and marker-dependent. In this context, the lack of significant changes in both erythrocyte and plasma MDA levels observed in our study may be interpreted in several ways. It should also be noted that erythrocyte and plasma MDA may reflect different aspects of oxidative stress and lipid peroxidation. Erythrocyte MDA is generally considered a marker of more stable and cumulative oxidative damage within cellular structures, whereas plasma MDA may reflect more dynamic and transient systemic oxidative processes. First, it may reflect the relatively mild to moderate severity of stroke in the studied cohort, in which oxidative damage is less extensive and more effectively controlled by endogenous antioxidant mechanisms [42,44]. Second, it may indicate that the selected time points (day 1 and day 8) do not capture the peak of lipid peroxidation, which may occur earlier or fluctuate dynamically during the acute and subacute phases of stroke [45]. Finally, the absence of significant MDA changes may also be consistent with the observed increase in SOD activity, potentially limiting downstream lipid peroxidation processes.

Taken together, these findings suggest that, in the early phase of AIS, lipid peroxidation markers such as MDA may not always reflect the full extent of OS, because MDA represents only one aspect of lipid peroxidation and may not fully capture the complexity of oxidative lipid damage, particularly in cohorts with less severe disease or when assessed at limited time points. This highlights the importance of integrating multiple OS markers, including both enzymatic and non-enzymatic components, to more comprehensively characterize redox imbalance in ischemic stroke.

In contrast to the enzymatic antioxidant parameters, our study demonstrated a significant decrease in both melatonin and 25(OH)D concentrations between day 1 and day 8 after stroke onset. These findings suggest that, alongside selective activation of enzymatic antioxidant defenses, AIS may be associated with a reduction in key systemic modulators of redox homeostasis.

Melatonin is widely recognized as a potent endogenous antioxidant with both direct and indirect neuroprotective properties [46]. In addition to its role as a free radical scavenger, melatonin enhances the activity of antioxidant enzymes and modulates mitochondrial function, thereby contributing to the maintenance of cellular redox balance [47,48]. Its decline observed in our study may therefore reflect increased utilization in response to OS, as well as a potential disruption of its physiological regulation during the acute phase of ischemic stroke. Reduced melatonin availability may consequently weaken the overall antioxidant capacity, particularly at the level of mitochondrial protection and free radical neutralization [49].

Similarly, vitamin D has been implicated in the regulation of both OS and inflammatory pathways, including modulation of NF-κB signaling and activation of Nrf2-dependent antioxidant responses [50]. A substantial body of literature has demonstrated an inverse relationship between serum 25(OH)D levels and stroke severity, with lower vitamin D concentrations associated with higher neurological deficit scores and worse functional outcomes [51,52,53]. Adequate vitamin D status has also been linked to improved recovery and reduced neurological impairment following ischemic stroke [53,54].

In contrast to these observations, our study focused on temporal changes in 25(OH)D concentrations rather than on their association with clinical outcome measures. Nevertheless, the significant decrease in 25(OH)D concentration over time observed in our cohort suggests that vitamin D may be dynamically involved in the systemic response to ischemic injury, rather than acting solely as a static baseline risk factor. Additionally, the lack of adjustment for potential confounding factors, such as age, comorbidities, or seasonal variation in vitamin D synthesis, may have further limited the ability to detect such associations.

Importantly, the concurrent decline in both melatonin and vitamin D may indicate a broader phenomenon of depletion of non-enzymatic antioxidant and regulatory reserves during the early post-stroke period. Unlike enzymatic antioxidants, which may be upregulated in response to OS, systemic modulators such as melatonin and vitamin D may be progressively consumed or dysregulated, leading to reduced capacity for long-term redox control and recovery support [55,56,57].

This observation may indicate that systemic antioxidant modulators are more vulnerable to depletion than enzymatic defenses, potentially representing a limiting factor in the overall redox response during the early phase of stroke.

**Figure 1:**
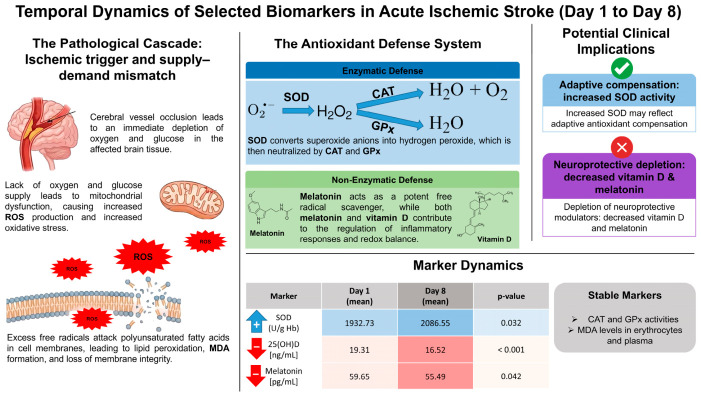
Schematic representation of the temporal dynamics of enzymatic and non-enzymatic antioxidant responses in acute ischemic stroke (AIS) between day 1 and day 8 after stroke onset. The left panel illustrates the pathophysiological cascade initiated by cerebral vessel occlusion, leading to oxygen and glucose deprivation, mitochondrial dysfunction, and increased production of reactive oxygen species (ROS), which promote lipid peroxidation of polyunsaturated fatty acids and the formation of malondialdehyde (MDA). The central panel presents the antioxidant defense system, including enzymatic components such as superoxide dismutase (SOD), catalase (CAT), and glutathione peroxidase (GPx). SOD catalyzes the conversion of superoxide anion (O_2_^•−^) into hydrogen peroxide (H_2_O_2_), which is subsequently detoxified by CAT to water (H_2_O) and oxygen (O_2_), or reduced to water by GPx. The non-enzymatic defense system includes melatonin and 25-hydroxyvitamin D (25(OH)D), which contribute to the regulation of inflammatory responses and redox balance. The lower panel summarizes the observed changes in selected biomarkers, showing a significant increase in SOD activity and significant decreases in 25(OH)D and melatonin, with no significant changes in CAT or GPx activity or MDA levels in erythrocytes and plasma. The right panel indicates potential clinical implications, suggesting that increased SOD activity may reflect adaptive antioxidant compensation, whereas decreased levels of melatonin and 25(OH)D may indicate depletion of systemic protective modulators during the early phase of acute ischemic stroke. In the marker dynamics panel, the blue upward arrow indicates an increase in SOD activity, the red downward arrows indicate decreases in 25(OH)D and melatonin concentrations, and the gray box indicates markers without significant temporal changes.

Taken together, these findings suggest that AIS is characterized not only by alterations in enzymatic antioxidant defenses but also by a decline in key systemic modulators of redox and inflammatory homeostasis. This dual pattern, selective enzymatic activation combined with depletion of non-enzymatic regulatory factors, may contribute to sustained oxidative imbalance and impaired adaptive response during the early stages of stroke. To integrate these findings, a schematic representation of the proposed redox response in acute ischemic stroke, including both enzymatic and non-enzymatic components, is presented in Figure 1.

From a translational perspective, the observed biomarker pattern may have potential clinical relevance because it suggests that early redox alterations in AIS are not limited to generalized oxidative damage, but may reflect an imbalance between compensatory enzymatic activation and depletion of systemic regulatory reserves. The increase in SOD activity, together with unchanged CAT and GPx activities, may indicate a partially effective but insufficiently coordinated antioxidant response, which could contribute to prolonged oxidative pressure and increased vulnerability of ischemic tissue to secondary injury. At the same time, the concomitant decrease in melatonin and 25(OH)D may suggest reduced systemic capacity to modulate oxidative and inflammatory processes involved in post-stroke recovery. Although these findings do not establish direct prognostic or therapeutic utility, they support the potential value of integrated redox profiling as a framework for identifying patients with different patterns of antioxidant adaptation and for guiding future studies on individualized supportive strategies in AIS.

Several limitations of the present study should be acknowledged when interpreting the results. First, the relatively small sample size may have limited the statistical power to detect subtle changes and associations, particularly in the context of heterogeneous OS responses. Additionally, the study population consisted predominantly of patients with mild to moderate stroke severity, as individuals with more severe neurological deficits were often unable to provide informed consent. This may have resulted in an underrepresentation of patients with more pronounced oxidative imbalance and could partly explain the lack of significant changes observed for some parameters, such as CAT, GPx, and MDA.

Furthermore, the analysis was restricted to two time points (day 1 and day 8), which limits the ability to fully characterize the dynamic and potentially non-linear temporal profile of OS and antioxidant responses in AIS, particularly during the earliest hours after stroke onset and reperfusion-related processes. It is possible that peak changes in certain biomarkers occur outside the selected observation window, particularly in the very early phase following ischemia or during later stages of recovery. Additionally, the implementation of standardized morning blood collection procedures, introduced to minimize circadian variability of biomarkers such as melatonin, may have further limited the number of eligible participants. In addition, formal multivariate analyses adjusting for potential confounders, such as age, comorbidities, or treatment modalities, were not performed due to the relatively small cohort size and the risk of model overfitting, which may have influenced the observed relationships between OS markers and clinical parameters.

Another important limitation is the absence of pre-stroke antioxidant status data and a matched healthy control group. Information on habitual dietary intake, long-term antioxidant supplementation, and pre-stroke levels of oxidative stress markers was not available. Therefore, we cannot fully determine whether the observed biomarker levels reflect stroke-related changes alone or are partly influenced by inter-individual baseline variability. However, the longitudinal within-subject design partially reduces this limitation by focusing on temporal changes within the same individuals between day 1 and day 8. Future studies should include matched control groups and, where feasible, information on baseline antioxidant status to better distinguish stroke-specific alterations from pre-existing redox differences.

Despite these limitations, the present study provides important insights into the temporal changes in both enzymatic and non-enzymatic components of the antioxidant system in AIS. In particular, the combined assessment of multiple OS markers, including enzymatic defenses and systemic modulators such as melatonin and vitamin D, allows for a more integrated evaluation of redox homeostasis than studies focusing on single biomarkers. This approach addresses a relevant gap in the current literature, where comprehensive, longitudinal analyses of both enzymatic and non-enzymatic antioxidant mechanisms remain limited. Although exploratory in nature, the longitudinal assessment of both enzymatic and non-enzymatic oxidative stress-related parameters provides clinically relevant insight into the dynamic redox response occurring during the early phase of AIS.

Given the exploratory nature of this study and the relatively small cohort size, the findings should be interpreted with caution and may be considered preliminary. Nevertheless, the observed pattern of selective enzymatic activation accompanied by a decline in systemic modulators suggests potentially important mechanisms underlying redox imbalance in the early phase of stroke, which warrant further investigation.

Future studies should aim to include larger and more clinically diverse patient populations, particularly incorporating individuals with severe stroke, in order to better characterize the full spectrum of OS responses. Expanding the temporal resolution through more frequent sampling could provide a more detailed understanding of the dynamic changes in redox biomarkers. Moreover, the integration of OS parameters with clinical outcomes, imaging data, and inflammatory markers may help to clarify their prognostic value and potential clinical utility. Inclusion of additional biomarkers associated with systemic oxidative and vascular stress, such as homocysteine, particularly in the context of highly prevalent vascular comorbidities including hypertension, as well as protein oxidation products or global antioxidant capacity indices, may further improve comprehensive characterization of redox imbalance in AIS. Finally, further research is needed to explore whether modulation of systemic factors, such as vitamin D or melatonin, could represent a potential therapeutic strategy to improve antioxidant capacity and support recovery in patients with AIS.

## 4. Materials and Methods

### 4.1. Study Design and Ethical Approval

This study was designed as a prospective observational clinical study evaluating OS markers in patients hospitalized due to AIS. The study followed a longitudinal within-subject design, with each patient serving as their own reference between day 1 and day 8 after stroke onset. Accordingly, the primary objective was to assess temporal changes in selected redox-related markers during the early post-stroke period rather than to compare absolute biomarker levels with those of a healthy control group. Blood samples were collected at two time points: on the 1st and 8th day after stroke onset.

Patients were consecutively recruited from the Department of Neurology, Antoni Jurasz University Hospital No. 1 in Bydgoszcz, Poland (9 Marii Skłodowskiej-Curie St., 85-094 Bydgoszcz, Poland). Laboratory analyses were performed at the Department of Medical Biology and Biochemistry, Faculty of Medicine, Ludwik Rydygier Collegium Medicum in Bydgoszcz, Nicolaus Copernicus University in Toruń, Poland (24 Karłowicza St., 85-092 Bydgoszcz, Poland).

The study protocol was approved by the Bioethics Committee of the Nicolaus Copernicus University in Toruń, functioning at the Collegium Medicum in Bydgoszcz, Poland (approval no. KB 559/2022, approved on 22 November 2022). The study was conducted in accordance with the principles of the Declaration of Helsinki. Written informed consent was obtained from all participants prior to their inclusion in the study.

### 4.2. Study Population

A total of 48 patients admitted to the Department of Neurology, Antoni Jurasz University Hospital No. 1 in Bydgoszcz, who met the inclusion criteria, were enrolled in the study.

The inclusion criteria were as follows: diagnosis of AIS, admission within 48 h of symptom onset, age ≥ 18 years, and the ability to provide informed consent.

The exclusion criteria included: hemorrhagic stroke, transient ischemic attack (TIA), acute coronary syndrome, infection at admission or within the last month, rheumatological diseases, active malignancy, surgery within the last 3 months, steroid therapy, pregnancy, contraindications to head magnetic resonance imaging (MRI), and inability to provide informed consent.

Stroke severity and clinical characteristics were assessed using the NIHSS, the ASPECTS, and the TOAST classification. NIHSS scores were categorized into four subgroups: 1–4 (mild), 5–15 (moderate), 16–20 (moderate–severe), and 21–42 (severe).

The baseline characteristics of the study population are presented in Table 2.

Information on medication use prior to hospitalization and during the hospital stay was collected from medical records and incorporated into the clinical characterization of the study cohort. Particular attention was paid to pharmacological agents known to influence oxidative stress and redox balance.

Before hospitalization, a proportion of patients were receiving standard therapies for cardiovascular and metabolic comorbidities, including statins, antihypertensive agents, and antidiabetic medications. During hospitalization, the use of these medications increased substantially, with the majority of patients receiving antiplatelet therapy, statins, and antihypertensive treatment in accordance with current clinical guidelines for acute ischemic stroke management.

Regarding supplementation, no patients reported the use of classical antioxidant supplements such as vitamin C, vitamin E, or N-acetylcysteine prior to admission. Melatonin use was not reported in any patient. Vitamin D supplementation was reported in a small number of patients before hospitalization, while additional patients received vitamin D during hospitalization. Other supplements, including folic acid, alpha-lipoic acid, and glutamine, were administered only in isolated cases.

A detailed summary of medication use before and during hospitalization is provided in Table 3.

### 4.3. Blood Collection and Biological Material

Venous blood samples were collected from the median cubital vein on day 1 and day 8 after stroke onset. Blood collection was performed by qualified medical personnel. For each time point, two types of tubes were used: a 6 mL tube containing ethylenediaminetetraacetic acid (EDTA) as an anticoagulant and a 6 mL tube without anticoagulant (for serum separation). Blood samples collected for research purposes were obtained during standardized morning sampling hours (approximately between 07:00 and 09:00) at both study time points. When morning collection at the initial study time point was not feasible, patients were not included in the longitudinal research sampling protocol. This procedure was implemented to minimize potential circadian variability of the analyzed biomarkers, particularly melatonin.

Immediately after collection, the samples were transported under controlled temperature conditions to the laboratory of the Department of Medical Biology and Biochemistry, Faculty of Medicine, Ludwik Rydygier Collegium Medicum in Bydgoszcz, Nicolaus Copernicus University in Toruń, Poland.

The blood samples were centrifuged at 6000× *g* for 10 min to separate plasma and cellular components (EDTA tubes) or serum and blood clot (tubes without anticoagulant). Following centrifugation, the obtained plasma and serum were aliquoted into microcentrifuge tubes and stored at −80 °C until further biochemical analysis.

### 4.4. Biochemical Analysis

The concentrations of thiobarbituric acid reactive substances (TBARS) in plasma (TBARSpl) and erythrocytes (TBARSer), as well as the activities of antioxidant enzymes, including SOD, CAT, and GPx, were determined using spectrophotometric methods with a Cary 60 UV–Vis spectrophotometer (Agilent Technologies, Mulgrave, VC, Australia) equipped with Cary WinUV Advanced Reads Application software, version 5. All reagents used for spectrophotometric analyses were purchased from Merck (Merck KGaA, Darmstadt, Germany).

The activity of SOD (EC 1.15.1.1) was determined based on the inhibition of adrenaline auto-oxidation to adrenochrome at alkaline pH, measured at 480 nm, according to the method of Misra and Fridovich [58]. One unit of SOD activity was defined as the amount of enzyme causing 50% inhibition of adrenaline oxidation.

The activity of CAT (EC 1.11.1.6) was determined by measuring the decomposition of hydrogen peroxide (H_2_O_2_) at 240 nm according to the spectrophotometric method of Beers and Sizer [59]. One unit of CAT activity was defined as the amount of enzyme catalyzing the decomposition of 1 μmol of H_2_O_2_ per minute at pH 7.0 and 25 °C.

The activity of GPx (EC 1.11.1.9) was determined using a coupled enzymatic assay, in which the oxidation of reduced GSH is accompanied by the conversion of NADPH to NADP^+^, monitored at 340 nm, according to the method of Paglia and Valentine [60]. One unit of GPx activity was defined as the amount of enzyme catalyzing the oxidation of 1 μmol of NADPH per minute.

Hb concentration was determined using the Drabkin method [61]. In this assay, Hb is oxidized to methemoglobin by potassium ferricyanide and subsequently converted to a stable cyanmethemoglobin complex. Absorbance was measured at 540 nm at room temperature.

The activities of CAT, SOD, and GPx were determined in erythrocytes and expressed as units per gram of Hb (U/g Hb).

The concentration of TBARS was determined according to the method of Buege and Aust [62], with modifications based on the procedure described by Esterbauer and Cheeseman [63]. The TBARS concentration in plasma was expressed as nanomoles of MDA per milliliter of plasma (nmol MDA/mL), whereas in erythrocytes it was expressed as nanomoles of MDA per gram of Hb (nmol MDA/g Hb). Since the TBARS assay mainly reflects MDA-related products, the results were expressed as MDA equivalents.

The concentrations of 25(OH)D and melatonin were determined in serum using commercially available enzyme-linked immunosorbent assay (ELISA) kits: IDK^®^ 25-OH Vitamin D ELISA (Immundiagnostik AG, Bensheim, Germany) and Melatonin ELISA Kit (Cloud-Clone Corp., Houston, TX, USA). All assays were performed in accordance with the manufacturers’ instructions. Absorbance was measured using a CLARIOstar multimode microplate reader (BMG LABTECH GmbH, Ortenberg, Germany). The concentrations of 25(OH)D and melatonin were expressed in nanograms per milliliter (ng/mL) and picograms per milliliter (pg/mL), respectively.

### 4.5. Statistical Analysis

Statistical analysis was performed using JASP software (version 0.19.3; JASP Team, University of Amsterdam, Amsterdam, The Netherlands).

Descriptive statistics were presented as mean with standard error of the mean (SEM), along with median and interquartile range (IQR), to comprehensively characterize the distribution of the data.

The normality of the distribution of paired differences between day 1 and day 8 was assessed using the Shapiro–Wilk test. The choice of statistical test was based on the distribution of paired differences rather than raw values.

Comparisons of biochemical parameters between day 1 and day 8 were performed using the paired Student’s *t*-test for variables with normally distributed paired differences. For variables that did not meet the assumption of normality, the non-parametric Wilcoxon signed-rank test was applied.

Correlations between oxidative stress-related biomarkers and NIHSS scores were assessed using Spearman’s rank correlation coefficient (Spearman’s rho) due to the relatively small sample size and non-normal distribution of NIHSS scores.

Effect sizes were calculated for all paired comparisons. For variables analyzed using the paired Student’s *t*-test, Cohen’s d was reported, whereas for variables analyzed using the Wilcoxon signed-rank test, rank-biserial correlation r was reported. Because the study had an exploratory observational design and the final sample size was determined by the number of eligible patients recruited during the study period, no a priori sample size calculation was performed. Instead, a sensitivity power analysis was conducted to determine the minimum effect size detectable with the available sample. Assuming a two-sided test, α = 0.05, power = 0.80, and an effective sample size of approximately 40 paired observations, the study was sensitive to detect effects of approximately |δ| ≥ 0.45, corresponding approximately to a moderate effect size. No formal correction for multiple comparisons was applied due to the exploratory nature of the study and the analysis of predefined biologically related biomarkers representing different components of the antioxidant defense system. Exact *p*-values and effect sizes are reported to allow transparent interpretation of the results.

A *p*-value of less than 0.05 was considered statistically significant.

## 5. Conclusions

In conclusion, our findings indicate that the early phase of AIS is characterized by a selective and temporally dynamic antioxidant response. This pattern is marked by increased SOD activity in the absence of corresponding changes in CAT and GPx, together with a significant decline in systemic modulators such as melatonin and 25(OH)D.

These results suggest that redox imbalance in AIS may arise not only from excessive ROS production but also from an asymmetric and potentially insufficient antioxidant response. The present findings highlight the importance of integrated assessment of both enzymatic and non-enzymatic components of the antioxidant system. Although the results should be interpreted with caution due to the exploratory nature of the study, they support a more comprehensive understanding of redox dysregulation and may contribute to the identification of novel biomarkers and potential therapeutic targets in AIS.

## Figures and Tables

**Table 1:** Temporal changes in enzymatic and non-enzymatic oxidative stress markers, including antioxidant enzyme activities (SOD, CAT, GPx), lipid peroxidation indices (MDA in erythrocytes and plasma), and selected endogenous modulators (melatonin and 25(OH)D), between day 1 and day 8 in patients with acute ischemic stroke.

Parameter	Measure	Day 1	Day 8	Statistic	*p*-Value	Effect Size
SOD [U/g Hb]	Mean	1932.73	2086.55	z = 2.14	0.032	r = 0.39
	SEM	64.73	99.15			
	Median	1815.73	1893.86			
	IQR	586.53	876.81			
CAT [×10^3^ U/g Hb]	Mean	403.19	415.30	t = 0.77	0.444	d = 0.12
	SEM	135.80	132.79			
	Median	392.90	425.00			
	IQR	136.43	118.40			
GPx [U/g Hb]	Mean	24.70	24.40	z = 0.72	0.477	r = 0.13
	SEM	0.92	0.92			
	Median	23.95	24.48			
	IQR	5.90	6.56			
MDAer [nmol MDA/g Hb]	Mean	182.96	187.15	t = 0.21	0.838	d = 0.03
	SEM	12.20	12.45			
	Median	197.13	198.14			
	IQR	126.22	152.85			
MDApl [nmol MDA/mL]	Mean	0.41	0.41	t = 0.10	0.922	d = 0.02
	SEM	0.01	0.01			
	Median	0.39	0.41			
	IQR	0.12	0.09			
Melatonin [pg/mL]	Mean	59.65	55.49	t = −2.10	0.042	d = −0.34
	SEM	3.90	3.27			
	Median	55.60	50.14			
	IQR	25.80	24.13			
25(OH)D [ng/mL]	Mean	19.31	16.52	z = −3.51	<0.001	r = −0.69
	SEM	2.11	1.81			
	Median	16.33	13.94			
	IQR	16.76	12.08			

Abbreviations used: 25(OH)D—25-hydroxyvitamin D; CAT—catalase; GPx—glutathione peroxidase; Hb—hemoglobin; IQR—interquartile range; MDAer—malondialdehyde in erythrocytes; MDApl—malondialdehyde in plasma; SEM—standard error of the mean; SOD—superoxide dismutase. For variables with normally distributed paired differences, the paired Student’s *t*-test was applied and results are reported with t statistics and Cohen’s d effect sizes. For variables with non-normally distributed paired differences, the Wilcoxon signed-rank test was applied and results are reported with z statistics and rank-biserial correlation r effect sizes. CAT activity is presented as ×10^3^ U/g Hb due to higher absolute activity values.

**Table 2:** Demographic and clinical characteristics of patients with acute ischemic stroke, including age, sex, BMI, vascular risk factors (hypertension, diabetes, prediabetes, dyslipidemia, smoking status), comorbidities (atrial fibrillation), stroke etiology (TOAST classification), radiological severity (ASPECTS), clinical severity (NIHSS at day 1 and day 8), and acute treatment modalities (thrombolysis, thrombectomy, no reperfusion therapy).

Characteristic	Mean ± SEM	Group	*n*	Percent [%]
Sex	–	Female	21	43.8
		Male	27	56.3
BMI [kg/m^2^]	27.05 ± 0.61	Normal	14	29.2
		Overweight	20	41.7
		Class I obesity	9	18.8
		Class III obesity	1	2.1
		Missing data	4	8.3
Age [years]	69.31 ± 1.59	<65	13	27.1
		65–74	19	39.6
		75–84	14	29.2
		≥85	2	4.2
Hypertension	–	Present	42	87.5
		Not present	6	12.5
Diabetes	–	Present	13	27.1
		Prediabetes	9	18.8
		Not present	26	54.2
Dyslipidemia	–	Present	22	45.8
		Not present	25	52.1
		Missing data	1	2.1
Smoking status	–	Current smoker	17	35.4
		Former smoker	1	2.1
		Non-smoker	29	60.4
		Missing data	1	2.1
Atrial fibrillation	–	Present	9	18.8
		Not present	34	70.8
		Missing data	5	10.4
Acute treatment	–	Thrombolysis	6	12.5
		Thrombectomy	2	4.2
		No reperfusion therapy	40	83.3
TOAST	–	Large-artery atherosclerosis	3	6.3
		Small-vessel occlusion	9	18.8
		Cardioembolism	8	16.7
		Stroke of other determined etiology	2	4.2
		Stroke of undetermined etiology	26	54.2
ASPECTS	–	10	31	64.6
		9	10	20.8
		8	4	8.3
		7	1	2.1
		6	1	2.1
		5	1	2.1
NIHSS (day 1)	–	1–4	19	39.6
		5–15	24	50.0
		16–20	1	2.1
		21–42	1	2.1
		Missing data	3	6.3
NIHSS (day 8)	–	1–4	34	70.8
		5–15	8	16.7
		Missing data	6	12.5

Abbreviations used: ASPECTS—Alberta Stroke Program Early CT Score; BMI—body mass index; NIHSS—National Institutes of Health Stroke Scale; SEM—standard error of the mean; TOAST—Trial of ORG 10172 in Acute Stroke Treatment. Mean ± SEM is reported only for continuous variables. A dash (–) indicates that mean ± SEM was not applicable for categorical variables.

**Table 3:** Pharmacological treatment administered before and during hospitalization.

Drug Category	Specific Medication	Drugs Pre-Hospitalization	Drugs During Hospitalization
Anticoagulant drugs	Vitamin K antagonists	1	0
	Direct oral anticoagulants	6	5
Antiplatelet drugs	Acetylsalicylic acid	8	46
	Clopidogrel	1	0
Antihypertensive drugs	Angiotensin-converting enzyme inhibitors/angiotensin II receptor blockers/mineralocorticoid receptor antagonists	28	33
	Calcium channel blockers	14	19
	Thiazide and thiazide-like diuretics	7	9
Antidiabetic drugs	Metformin	13	5
	Sulfonylurea derivatives	5	0
	Insulin	5	17
	Others	3	1
Lipid-lowering drugs	Statin	16	44
	Ezetimibe	1	0
Other	Vitamin D	2	9
	Beta blockers	18	22

Data are presented as the number of patients receiving each medication category. Medications administered during hospitalization refer to treatments received within the first 8 days of hospital stay.

## Data Availability

The data presented in this study are available on request from the corresponding author. The data are not publicly available due to privacy and ethical restrictions related to the use of clinical data from human participants.
